# Delayed Rewarming Thrombocytopenia (DRT): A Temperature-Dependent Platelet Aggregation Disorder

**DOI:** 10.3390/hematolrep18030035

**Published:** 2026-05-27

**Authors:** Ian Joseph Cohen

**Affiliations:** 1 The Rina Zaizov Department of Pediatric Hematology Oncology, The Schneider Children’s Hospital of Israel, Petah Tikva 4920235, Israel; icohen@tauex.tau.ac.il; 2 The Sackler Faculty of Medicine, Tel Aviv University, Ramat Aviv, Tel Aviv 6997801, Israel

**Keywords:** delayed rewarming thrombocytopenia, hypothermia, platelet aggregation, heparin, aspirin, alcohol

## Abstract

Below 32 °C, the second irreversible stage of platelet aggregation is absent, causing augmentation of the first reversible stage of platelet aggregation and adhesion. During rewarming, de-aggregation occurs; however, in the presence of adequate ADP (adenosine diphosphate), the second stage of aggregation occurs, leading to delayed rewarming thrombocytopenia (DRT). Erythrocytes leak ADP in sufficient amounts by 24 h to cause DRT. This is prevented by rewarming within 24 h. Heparin before hypothermia prevents platelet adhesion, as does alcohol, which also blocks the second phase of aggregation. Aspirin blocks the second phase of aggregation, and platelet infusions, stored without erythrocytes, are an effective therapy. DRT explains rewarming deaths in NCI (neonatal cold injury).

## 1. Introduction

The case described by Henoch in 1889 [1] as “oedema of the newborn” seems to be the first report of the condition that Nassau referred to in 1948 as neonatal cold injury [2]. Those newborns with the condition are rosy-cheeked, quiet, do not cry, and only when it is realized that they do not feed and are cold to the touch is the diagnosis recognized. The realization that the condition was due to a low ambient temperature followed the availability of both thermometers and low-reading clinical thermometers. Thrombocytopenia, known to occur in hypothermia since 1938 [3], had not been considered significant, as the platelet count returned to normal upon rewarming [4]. Hessel demonstrated that while thrombocytopenia depended on cooling from 30 °C to 20 °C, the platelet count normalized on rewarming [4]. Isotope studies showed that the platelets were “sequestrated” in the sinusoids of the liver (and to a smaller amount in the spleen) and that the hypothermic episode did not affect platelet survival。 The platelets that reappeared in the bloodstream were shown to be the same platelets that had been sequestered in the liver [4,5]. Thrombocytopenia in fatal cases had been assumed to be due to DIC in the absence of any other explanation [6,7,8]. The reason why some infants who became hypothermic due to environmental conditions develop neonatal cold injury (NCI) and die while others survive [9] was unclear before my discovery in 1987 that the second stage of platelet aggregation and the release reaction do not occur below 32 °C [10]. There was until then no consensus as to whether the recommended treatment should be rapid or slow rewarming [11], nor had any prognostic indicators been found that differentiated survivors from fatal cases [9]. It was known that infants who died often had evidence of a bleeding diathesis, especially bleeding into the lungs, brain, and gut, and thrombocytopenia had been noted by several researchers [12,13]. Racine and Jarou reported that they had seen a group of infants who, on being slowly rewarmed to 32–33 °C, became active and developed a normal pulse and respiration. At this stage, they suddenly developed profuse bleeding into their lungs and gastrointestinal tract, and all died with a low platelet count [14].

## 2. Platelet Activation in Normothermia and Hypothermia

**Figure 1:**
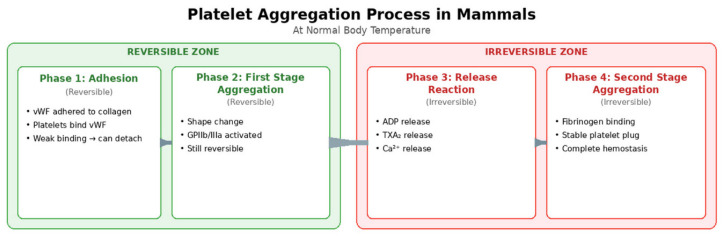
Platelet aggregation process in mammals at normal body temperature. The reversible stages of adhesion and the first stage of aggregation progress to the second, irreversible stage of aggregation, followed by the release reaction of dense granules and alpha granules.

Understanding the pathophysiological basis of the difference in platelet activation below and above 32 °C requires a review of platelet activation (Figure 1). At normal body temperature, platelet activation occurs in several stages. The first stage is adhesion, which occurs through the von Willebrand (vWF)–collagen interaction, in which platelets adhere to fibrinogen, collagen, and the vascular subendothelium. This is followed by the (reversible) first stage of platelet aggregation, then by the (irreversible) second stage of platelet aggregation with the release of dense granules [15]. Similarly, platelet alpha granules that contain large protein cargos such as fibrinogen are secreted on platelet activation.

**Figure 2:**
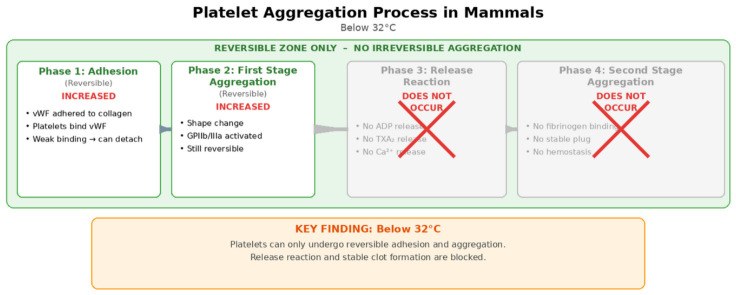
Platelet aggregation process in mammals below 32 °C (in vitro and in vivo). Following adhesion and the first stage of platelet aggregation, the second stage of aggregation and the release reaction do not occur. This leads to augmentation of the first stage of platelet aggregation, which remains reversible when platelets are rewarmed to 32 °C.

Examination of the aggregation curve of human platelets at 24 °C in the presence of ADP showed a more pronounced aggregation curve than at 37 °C (Figure 2). Further examination showed that the aggregation was reversible on rewarming, and that it was possible to demonstrate that the second phase of aggregation had not occurred, nor was the dense granule release reaction observed [10]. This block in the aggregation sequence results in increased platelet adhesion and a greater magnitude of the first reversible stage of platelet aggregation (first-stage platelet hyper-aggregation). In other words, the absence of the second stage of aggregation results in the first stage being augmented, since it cannot continue to the second stage of activation [16]. This is manifested clinically as thrombocytopenia that becomes more pronounced as the temperature drops [12], and platelet disaggregation occurs when platelets are rewarmed [5]. Multiple lines of evidence from different methods (Table 1) support the finding that below 32 °C, platelets undergo only reversible first-stage aggregation without progressing to irreversible second-stage aggregation or dense granule release. This dysfunction is completely reversible upon rewarming.

## 3. Hypothermic Platelet Aggregation in the Presence of Larger Quantities of ADP

Aggregometer studies of platelets with ADP (adenosine diphosphate) confirm that although the second stage of platelet aggregation is absent below 32 °C, on rewarming, the platelets de-aggregate and reappear in the bloodstream [10]. However, when hypothermic platelets are subjected to higher amounts of ADP in aggregometer studies, rewarming does not result in de-aggregation: instead, a sudden, massive second stage of aggregation (second-stage platelet hyper-aggregation) occurs, accompanied by the release of dense bodies [16]. In hypothermic conditions, it has been shown in vivo that ADP leaks from erythrocytes present in blood [21,22], and after 24 h of hypothermia, the levels of ADP would seem to be high enough to cause on rewarming the second stage of aggregation, leading in vivo to potentially fatal thrombocytopenia [16].

This has been designated DRT (delayed rewarming thrombocytopenia) to differentiate it from DIC [16].

Although the potentially reversible drop in platelet count during hypothermia can cause internal bleeding, the major danger of fatal bleeding is during rewarming after a hypothermic duration of 24 h (prolonged hypothermic duration—PHD) [16] when the sudden, dramatic, severe thrombocytopenia caused by the reappearance of the second phase of platelet activation occurs.

## 4. Differentiation of DRT from DIC 

In the past, in the absence of any other explanation, researchers had suggested that in fatal cases, thrombocytopenia was related to DIC [6,7,8]. However, this did not explain why neonates undergoing hypothermic surgery did not suffer from this complication. Chad and Gray suggested that this could have been due to routine heparinization [6].

Reevaluation of the reports of DIC in cases of NCI shows several reasons that DIC should no longer be considered as being associated with hypothermia [23].

The case reports that concluded there was a link between DIC and NCI do not stand up to close examination. (Table 2).

Some cases of hypothermia victims thought to have been suffering from DIC have not been reported accurately, and others that do seem to have suffered from DIC had alternative reasons for this complication.

Examples of such problematic reports [23] include the case report by Mahajan et al., cited at least 58 times [26], of a patient who received treatment with fluid heated to 43 °C. Hyperthermia explains the DIC in this case [27]. Other cases they cited as having elevated FDP levels did not in fact report such data, and they included a series without evidence of DIC. In that study, the three patients in whom FSP was measured had normal levels < 10 μ/mL [23]. The case report by Mahood and Evans [25] in which DIC was documented was of a complicated case of hypothermia with pancreatitis, which in itself is known to cause DIC. Other problematic case reports used clotting tests that were not performed at 37 °C. Reed showed [24] that if clotting tests were performed at the temperature of the examined rats, the results were abnormal for aPTT, PT, and TT, but were potentially reversible and became normal when the same blood samples were warmed to 37 °C. This seriously questions the diagnosis of DIC in case reports where clotting studies were performed at room temperature.

DRT differs from DIC in that it only occurs after prolonged hypothermic duration (PHD) [23]. In cases of DIC, it was possible to show that before diagnosis, the initial changes of the clotting factors thrombin–antithrombin III complex, plasmin–alpha 2 plasmin-inhibitor complex (PIC), and FDF-D-dimer levels became higher before changes in platelet counts, FDP, PT, and fibrinogen occurred. This demonstrates that thrombocytopenia is not the initiator of DIC, but a secondary occurrence [28], differing greatly from DRT in which thrombocytopenia is often the first and only abnormality.

Today, there is a consensus that DIC is to be suspected in the presence of thrombocytopenia, low fibrinogen, a finding of FDP (fibrin degradation products), high FSP (fibrin split products), or D-dimers, abnormal PT (prothrombin time), thrombin time (TT), and activated partial thromboplastin time (aPTT) [29]. However, these clotting factors, apart from FSP, FDP, and D-dimers, are affected by hypothermia [23]. Thus, the initial reports of abnormal clotting studies in hypothermia, as mentioned above, were artifacts of the temperatures at which the tests were performed. The realization that DRT, and not DIC, is the potentially fatal complication in NCI is fundamental to the development of different therapeutic approaches based on the pathophysiology involved. Minimal additional interventions, such as including the platelet count in the blood tests performed during rewarming, have the potential to significantly improve outcomes by uncovering significant thrombocytopenia before clinical bleeding occurs.

## 5. Clinical Manifestation of DRT in Neonatal Cold Injury

In a small proof-of-concept study of seven neonates with cold injury [18], five (71%) developed thrombocytopenia during the rewarming period in the first 24 h of hospitalization. One neonate died after developing severe thrombocytopenia. Another neonate survived following treatment with platelet transfusion despite a platelet nadir of 8000/μL. The thrombocytopenia that deepened during rewarming was consistent with DRT, suggesting that they had been rewarmed to 32 °C after a hypothermic duration of at least 24 h.

## 6. Avoidance of DRT

**Figure 3:**
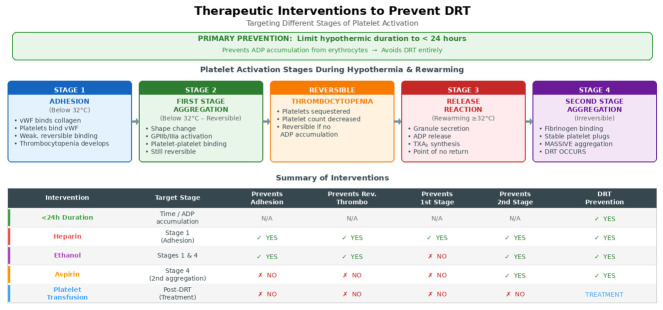
Therapeutic interventions at each stage of platelet activation to prevent DRT.

Several Interventions Have Been Shown to Prevent or Treat DRT (Figure 3). 

### 6.1. Early Rewarming (<24 h)

Although it has been accepted practice to measure the time to rewarming from rescue or hospitalization or start of rewarming, it has been suggested that it is more logical to measure the time that passed since the initiation of hypothermia (hypothermic duration). Although this is often not known accurately, it is usually possible to estimate based on the last known time the victim was seen before the hypothermia. A hypothermic duration of less than 24 h will avoid any danger of DRT developing. This can be achieved if the infant is seen before DRT occurs and treated by rapid rewarming before this time limit is reached. This intervention exploits the temporal requirement for ADP accumulation, with rewarming initiated before erythrocyte ADP leakage reaches critical levels sufficient to trigger second-stage hyper-aggregation. This observation is supported by the finding by Zingg [30] that in rabbits, rapid rewarming was more effective than slow rewarming only in the first 24 h. This explains why some have found rapid rewarming more effective than slow rewarming, while others have not. The paradoxical good outcome in attempted infanticide is now explained by the fact that early diagnosis of salvageable infants made within hours of birth enables rewarming to be performed within 24 h [31]. There have been dramatic case reports of infants that survived, such as the 5 h-old baby found with a rectal temperature of 16.2 °C in cardiac arrest and wrapped in a garbage bag. The fact that she had been inside a freezer for four hours and survived without any evidence of sequelae supports the suggestion that DRT based on the length of the hypothermic duration is the only significant cause of death in these neonates [32]. This is in contrast to the cases of NCI from environmental causes in whom the diagnosis is often delayed since the babies are, as mentioned above, rosy-cheeked, quiet, do not cry, and only when it is realized that they do not feed and are cold to the touch is the diagnosis made, often after many hours, thereby preventing rewarming before 24 h of hypothermic duration have passed.

### 6.2. Prevention of Platelet Adhesion: Heparin

Heparin effectively prevents hypothermia-induced thrombocytopenia when administered before the onset of hypothermia by blocking platelet adhesion to fibrinogen, collagen, and the vascular subendothelium. By preventing the initial adhesion step, heparin interrupts the entire activation sequence, preventing both sequestration and subsequent DRT. However, heparin administered after adhesion has already occurred (i.e., after hypothermia onset) is ineffective in preventing DRT. Heparin prevents the thrombocytopenia seen during hypothermia by blocking platelet adhesion to fibrinogen, collagen, and the vascular subendothelium [33,34]. Initial attempts at hypothermic neurosurgery were abandoned because of bleeding until Wensel and Bigelow were able to show that heparin prevented the thrombocytopenia [35]. Since heparin is routinely used with cardiopulmonary bypass, DRT is no longer a significant problem in hypothermic cardiac surgery. However, it is effective only if administered before hypothermia is induced before platelet adhesion has occurred. Under hypothermic conditions, heparin blocks adhesion; however, after adhesion has occurred, it will not prevent platelet activation and therefore is not a therapeutic option for hypothermia treatment. The thrombocytopenia seen with hypothermia will not occur if heparin is given before hypothermia. The aggregation sequence will not continue, since the prevention of adhesion will interrupt aggregation and the subsequent thrombocytopenia.

### 6.3. Inhibition of Second-Stage Aggregation: Aspirin

Aspirin selectively blocks the second stage of platelet aggregation through inhibition of thromboxane A_2_ formation. Aspirin-treated samples do not prevent the initial platelet count decline during hypothermia (due to intact adhesion and first-stage aggregation). Still, they prevent DRT during rewarming by blocking the irreversible second-stage hyper-aggregation. Notably, despite the absence of second stage aggregation capability, clinical bleeding does not occur, demonstrating adequate hemostatic function with first-stage aggregation alone. Aspirin affects platelet function by blocking the second stage of platelet aggregation through inhibition of thromboxane A_2_ formation [36] not affecting adhesion or the first stage of aggregation. It therefore does not prevent the drop in the platelet count seen during hypothermia due to “adhesion” and the first stage of aggregation (seen as sequestration of platelets in liver sinusoids) [19]. When platelets are rewarmed to 32 °C, DRT will not occur, and although the platelets cannot undergo the irreversible second stage of aggregation, clinical bleeding is not seen.

### 6.4. Prevention of Platelet Adhesion and Inhibition of Second-Stage Aggregation: Alcohol

Ethanol demonstrates dual mechanisms of platelet inhibition: blocking platelet adhesion to fibrin and inhibiting thromboxane A_2_ formation (similar to aspirin). This dual action prevents both the initial thrombocytopenia during hypothermia and the subsequent development of DRT during rewarming. Ethanol has been shown to prevent thrombocytopenia in hypothermia by inhibiting platelet adhesion to fibrin [37], and so prevents thrombocytopenia in hypothermia [38]. It also modifies platelet function in a similar way to that seen with aspirin by inhibition of thromboxane A_2_ formation [33], and so will prevent DRT on rewarming. A similar effect has been reported with other drugs, especially benzodiazepines [36], and may well be true of other ADP-receptor antagonists such as clopidogrel, ticagrelor, and prasugrel [39].

### 6.5. Therapeutic Intervention: Platelet Transfusion

Platelet transfusions successfully treat established DRT [18]. The efficacy of transfused platelets was attributed to their storage conditions: platelets for transfusion are stored separately from erythrocytes and therefore have not been exposed to ADP. These “ADP-naïve” platelets maintained normal function and provided therapeutic benefit despite the ongoing hypothermic/rewarming stress in the recipient.

## 7. DRT in Adults

Although there is no published evidence of proven DRT in adult cases, there are specific situations when adults who have suffered from prolonged hypothermia have been associated with a poorer outcome than other similar cases. It is not suggested that DRT has been proven responsible for the difference in outcome, but rather that these reports are compatible with the possibility of DRT being involved.

1. Cases of urban hypothermia found indoors are less likely to come to the attention of rescuers promptly and may not be discovered early enough to benefit from rewarming before being subject to DRT. One study showed that the survival of 45 patients discovered indoors was significantly worse than that of 35 found outdoors (*p* > 0.0001) [40].
2. Two groups of healthy teenagers who were both treated in hypothermic cardiac arrest with extracorporeal circulation differed in their outcomes. One significant difference was hypothermic duration. After a boating accident, all seven of the first group who were flown to the hospital by helicopter within 4.5 h survived [41], but there were only two survivors in the second group of eight adolescents and two middle-aged teachers trapped by a snowstorm rescued after 2 to 3 days [42].

## 8. Conclusions

The delineation of DRT and the knowledge that ADP leaks from erythrocytes in the blood enables this syndrome to be prevented, easily diagnosed, and treated. In neonatal cold injury, the only clinical intervention required to prevent DRT is to estimate the time the hypothermic episode started and to perform platelet counts in addition to the routine blood tests performed during rewarming. This is not routinely performed, if the relevant studies of neonatal cold injury are to be believed, probably because the known thrombocytopenia that occurs during hypothermia is considered self-limiting and has been assumed to disappear on rewarming. When platelet counts drop during rewarming, aspirin and alcohol can provide a preventive option. Early rewarming exploits the temporal kinetics of ADP accumulation, and platelet transfusion offers a rescue therapy even after DRT has developed. The choice of intervention can be tailored to the clinical scenario, timing of presentation, and available resources.

Understanding the pathophysiology of DRT also clarifies why traditional approaches based on the DIC paradigm were unsuccessful. Anticoagulation with heparin after hypothermia onset, fresh frozen plasma transfusions, and DIC-directed therapies do not address the fundamental problem of ADP-mediated platelet hyper-aggregation during rewarming. The paradigm shift from DIC to DRT thus represents not merely a semantic distinction, but a fundamental reconceptualization that enables rational, mechanism-based therapeutic strategies.

## 9. Limitations/Future Directions

Much of the data presented have been observational, and further experimental data are needed to confirm the conclusions reached. Following platelet counts during rewarming and noting the hypothermic duration are critical requirements for any challenge to the findings presented here. Currently, there is much speculation as to the possibility of prolonged hypothermia being utilized to enable survival during the time required to reach distant planets. If problems such as DRT are not appreciated and overcome, this will prevent the development of such approaches. The technique of hypothermic preservation of terminally sick cancer patients in the hope that in the future they could be resuscitated and benefit from treatments not yet available would present a similar challenge.

## Figures and Tables

**Table 1:** Key evidence for absence of second-stage platelet aggregation below 32 °C.

Evidence Type	Methodology	Key Finding
Functional assay	Aggregometry	Aggregation was reversible on rewarming. No second stage was seen with increasing ADP amounts. The second stage was demonstrated only on warming with adequate ADP [10].
Biochemical marker	Luciferase ATP release assay	Luciferase showed release reaction paralleled second-stage aggregation only during rewarming, not at hypothermic temperatures [10,17].
Inhibitor study	Pharmacologic intervention	Second-stage aggregation is prevented by aspirin during rewarming, but not needed at cold temperatures where the second stage does not occur [17,18].
Dose– response study	Agonist concentration study	Larger quantities of ADP at hypothermia cause an augmented first-stage response, but still no second-stage aggregation until rewarming occurs [10,17].
In vivo observation	Histopathology	Activated platelets without fibrin formation in hypothermic patients’ spleens [19]
Clinical study	Surgical/clinical setting	Reversible platelet dysfunction; function restored upon rewarming to 37 °C [20]

Summary: Multiple lines of evidence demonstrate that below 32 °C, platelets undergo only reversible first-stage aggregation without progression to irreversible second-stage aggregation or dense granule release. This dysfunction is completely reversible upon rewarming.

**Table 2:** Evidence of how DRT differs from DIC.

Feature	DRT (Delayed Rewarming Thrombocytopenia)	DIC (Disseminated Intravascular Coagulation)
**Definition**	Complications of prolonged hypothermia and rewarming [16]	Widespread hypercoagulable state causing micro/macrovascular clotting and organ dysfunction
**Mechanism**	Second-stage platelet aggregation blocked below 32 °C, causing first-stage hyper-aggregation [10,16]	Increased platelet aggregation and coagulation factor consumption with widespread fibrin deposition
**Precipitating Event**	Occurs after ≥24 h of hypothermia [16]	Acute complication of severe sepsis, malignancy, trauma, or placental abruption
**Temperature Dependence**	Second-stage aggregation is absent below 32 °C [10]	No temperature dependence
**Primary Defect**	Rewarmed platelets undergo sudden second-stage irreversible aggregation [16]	Dysregulated coagulation and fibrinolysis with widespread clotting and bleeding
**Thrombocytopenia**	Reversible thrombocytopenia [4]	Most frequent laboratory abnormality; irreversible without treatment
**Coagulation Factors**	Preserved; not consumed [23]	Variably decreased due to activation and consumption
**PT/PTT**	May be abnormal due to temperature effects [24]	Increased due to widespread activation and consumption
**D-dimer**	Normal or mildly elevated [23]	Invariably elevated, often dramatically (>4000 ng/mL)
**Clinical Presentation**	Massive pulmonary hemorrhage and bleeding after rewarming [14,18]	Venous thrombosis (slow DIC) or bleeding (rapid DIC)
**Timing**	Sudden bleeding after ≥24 h hypothermia [16]	Variable, depends on underlying cause
**Prevention**	Blocks second-stage platelet aggregation before/during hypothermia [16,22,23,25]	Treat underlying cause
**Treatment**	Blocks second-stage aggregation mechanism; platelet transfusion [23]	Treat cause of DIC.
**Pathophysiology**	Platelet-specific aggregation disorder [16]	Excessive thrombin generation with microvascular thrombosis and factor consumption
**Key Distinguishing Feature**	Reversible thrombocytopenia [4,5,23]	Consumption coagulopathy with prolonged PT/PTT, decreased platelets/fibrinogen, elevated FDPs

## Data Availability

No new data were created or analyzed in this study. Data sharing is not applicable to this article.

## Abbreviations

DRT	delayed rewarming thrombocytopenia
NCI	neonatal cold injury
DIC	disseminated intravascular coagulation
PHD	prolonged hypothermic duration
ADP	adenosine diphosphate
vWF	von Willebrand factor
GPIIb/IIIa	glycoprotein IIb/IIIa
TXA_2_	thromboxane A_2_
FDP	fibrin degradation products
FSP	fibrin split products
PT	prothrombin time
TT	thrombin time
PIC	plasmin-inhibitor complex
COX	cyclooxygenase
FFP	fresh frozen plasma
aPTT	activated partial thromboplastin time

## References

[1] Henoch E.H., Thomson J. (1889). Lectures in Children’s Diseases: A Handbook for Practitioners and Students.

[2] Nassau E. (1948). Kaltenschaden im Subtropischen Klima. Ann. Paeditr..

[3] Tocantins L.M. (1938). The Mammalian blood platelet in health and disease. Medicine.

[4] Hessel E.A., Schmer G., Dillard D.H. (1980). Platelet kinetics during deep hypothermia. J. Surg. Res..

[5] Villalobos T.J., Adelson E.A., Riley P.A., Crosby W.H. (1958). A cause of thrombocytopenia and leukopenia that occurs in dogs during deep hypothermia. J. Clin. Investig..

[6] Chad M.A., Gray O.P. (1972). Hypothermia and coagulation defects in the newborn. Arch. Dis. Child..

[7] Thomas D.B. (1974). Detection and treatment of severe coagulation disturbances in the neonatal period. Med. J. Aust..

[8] Johansson B.W., Nilsson I.M. (1964). The effect of heparin and Epsilon-aminocaproic acid on the coagulation of hypothermic dogs. Acta Physiol. Scand..

[9] Bower B.D., Jones L.F., Weeks M.M. (1960). Cold injury in the newborn. Br. Med. J..

[10] Cohen I.J., Fuchs J., Kaplinski C., Krugliak J., Stark B., Vogel R., Weinberger I., Rotenberg Z., Agmon J., Jerushalmyi Z. (1987). Room temperature ADP induced first stage hyperaggregation of human blood platelets: A previously undescribed phenomenon and its relationship to spontaneous cold induced platelet aggregation. Br. J. Haematol..

[11] Sofer S., Yagupsky P., Hershkowitts J., Bearman J.E. (1986). Improved outcome of hypothermic infants. Pediatr. Emerg. Care.

[12] Mann T.P., Elliot R.I.K. (1957). Neonatal Cold Injury. Lancet.

[13] Cullic S. (2005). Cold injury syndrome and neurodevelopmental changes in survivors. Arch. Med. Res..

[14] Racine J., Jarjoui E. (1982). Severe hypothermia in infants. Helv. Paediatr. Acta.

[15] Li Z., Delaney M.K., O’Brien K.A., Du X. (2010). Signaling During Platelet Adhesion and Activation. Arterioscler. Thromb. Vasc. Biol..

[16] Cohen I.J. (2024). Delayed rewarming thrombocytopenia: A suggested preventable and treatable cause of rewarming deaths. J. Pediatr. Hematol. Oncol..

[17] Cohen I.J. (1991). Room temperature ADP-induced first stage hyper aggregation of human platelets: The cause of rewarming deaths in neonatal cold injury. Pediatr. Hematol. Oncol..

[18] Cohen I.J., Amir J., Gedaliah A., Rachmal A., Gorodisher R., Zaizov R. (1984). Thrombocytopenia of neonatal cold injury. J. Pediatr..

[19] Horioka K., Tanaka H., Okaba K., Yamada S., Ishii N., Motomura A., Inoue H., Alkass K., Druid H., Yajima D. (2021). Hypothermia causes platelet activity in the human spleen. Thromb. Res..

[20] Valeri C.R., Feingold H., Cassidy G., Ragno G., Khuri A., Altshule M.D. (1987). Hypothermia-induced reversible platelet dysfunction. Ann. Surg..

[21] Knofler R., Weissbach G., Kuhlisch E. (1997). Release of adenosine triphosphate by adenosine diphosphate in whole blood and in erythrocyte suspensions. Am. J. Hematol..

[22] Straub A., Krajewski S., Hohmann J.D., Westein E., Jia F., Bassler N., Selan C., Kurz J., Wendel H.P., Dezfouli S. (2011). Evidence of platelet activation at medically used hypothermia and mechanistic data indicating ADP as a key mediator and therapeutic target. Arterioscler. Thromb. Vasc. Biol..

[23] Cohen I.J. (2025). Delayed Rewarming Thrombocytopenia Not Disseminated Intravascular Coagulation, is the Cause of Rewarming Deaths in Neonatal Cold Injury. J. Pediatr. Hematol. Oncol..

[24] Reed R.L., Johnston T.D., Hudson J.D., Fischer R.P. (1992). The disparity between hypothermic coagulopathy and clotting studies. J. Trauma..

[25] Mahood J.M., Evans A. (1978). Accidental hypothermia, disseminated intravascular coagulation, and pancreatitis. N. Z. Med. J..

[26] Mahajan S.L., Myer T.J., Baldini M.G. (1981). Disseminated Intravascular Coagulation following hypothermia. JAMA.

[27] Diehl K.A., Crawford E., Shinko P.D., Tallman R.D., Oglesbee M.J. (2000). Alterations in hemostasis in the canine model. Am. J. Hematol..

[28] Wada H., Minamikawa K., Wakita Y., Nakase T., Kaneko T., Ohiwa M., Tamaki S., Deguchi A., Mori Y., Deguchi K. (1993). Hemostatic study before onset of Disseminated Intravascular Coagulation. Am. J. Hematol..

[29] Taylor F.B.J., Toh C.H., Hoots W.K., Wada H. (2001). Scientific Committee on Disseminated Intravascular Coagulation (DIC) of the International Society on Thrombosis and Hemostasis (ISTH)Towards definition, clinical and laboratory criteria for disseminated intravascular coagulation. Thromb. Hemost..

[30] Zingg W. (1969). Fast and slow warming after acute and prolonged hypothermia. J. Trauma..

[31] Cohen I.J. (2026). Unexpected survival of neonates after attempted hypothermic infantocide. Forensic Sci. Med. Pathol..

[32] Thompson D.A., Anderson N. (1994). Successful resuscitation of a severely hypothermic neonate. Ann. Emerg. Med..

[33] Davies J.A., Menys V.C. (1978). Inhibition by Heparin of platelet adhesion to collagen and sub-endothelium. Clin. Sci. Mol..

[34] Niimi Y., Ishiguro Y., Nakata Y., Morita S., Yamane S. (2021). Platelet adhesion to heparin-coated oxygenator fibers under in vitro conditions: Impact of temperature. ASAIO J..

[35] Wensel R.H., Bigelow W.G. (1959). The use of Heparin to minimize thrombocytopenia and bleeding tendency during hypothermia. Surgery.

[36] Vane J.R. (1971). Inhibition of Prostaglandin Synthesis as a mechanism of action for Aspirin -like drugs. Nat. New Biol..

[37] de Lange D.W., Sholman W.L.G., Kraaijenhagen R.J., Akkerman J.W.N., Van de Wiel A. (2004). Alcohol and polyphenolic grape extract inhibit platelet adhesion in flowing blood. Eur. J. Clin. Investig..

[38] Renaud S.C., Ruf J.-C. (1996). Effects of Alcohol on Platelet Function. Clin. Chim. Acta.

[39] Romstedt K., Akbar H. (1985). Benzodiazepines inhibit platelet activation: Comparison of the mechanism of antiplatelet actions of fluzepam and diazepam. Thromb. Res..

[40] Roeggla M., Holzer M., Roeggla G., Frossard M., Wagner A., Laggner A.N. (2001). Prognosis of accidental hypothermia in the urban setting. J. Intensive Care Med..

[41] Wanscher M., Agersnap L., Ravn J. (2012). Outcome of accidental hypothermia with or without cardiac arrest. Resuscitation.

[42] Hauty M.G., Esrig B.C., Hill J.G., Long W.B. (1987). Prognostic factors in severe accidental hypothermia: Experience from the Mt Hood Tragedy. J. Trauma.

